# Navigating the COVID-19 Crisis to Sustain Community-Based Malaria Interventions in Cambodia

**DOI:** 10.9745/GHSP-D-20-00528

**Published:** 2021-06-30

**Authors:** Mitra Feldman, Lieven Vernaeve, James Tibenderana, Leo Braack, Mark Debackere, Htin Kyaw Thu, Prudence Hamade, Koung Lo

**Affiliations:** a Research scientist, Monteverde, Costa Rica.; b Malaria Consortium, Phnom Penh, Cambodia.; c Malaria Consortium, London, United Kingdom.; d Malaria Consortium Asia Regional Office, Bangkok, Thailand.; e Provincial Health Department, Preah Vihear Province, Ministry of Health of Cambodia, Preah Vihear, Cambodia.

## Abstract

Cambodia has made impressive progress in reducing malaria trends and, in 2018, reported no malaria-related deaths for the first time. However, the coronavirus disease (COVID-19) pandemic presents a potential challenge to the country's goal for malaria elimination by 2025. The path toward malaria elimination depends on sustained interventions to prevent rapid resurgence, which can quickly set back any gains achieved.

Malaria Consortium supported mobile malaria workers (MMWs) to engage with target communities to build acceptance, trust, and resilience. At the start of the pandemic, Malaria Consortium conducted a COVID-19 risk assessment and quickly developed and implemented a mitigation plan to ensure MMWs were able to continue providing malaria services without putting themselves or their patients at risk. Changes in malaria intervention coverage and community uptake have been monitored to gauge the indirect effects of COVID-19. Comparisons have been made between output indicators reported in 2020 and from the same month-period of the previous year.

In general, malaria service intervention coverage and utilization rates did not decline in 2020. Rather, the reported figures show there was a substantial increase in service utilization. Preliminary internal reviews and community meetings show that despite a heightened public risk perception toward COVID-19, malaria testing motivation has been well sustained throughout the pandemic. This may be attributable to proactive program planning and data monitoring and active engagement with the communities and the national authorities to circumvent the indirect effect of COVID-19 on intervention coverage in Cambodia during the pandemic.

## Abstract (precis)

Despite the impacts of an unforeseen concomitant disaster such as COVID-19, malaria elimination efforts were able to continue because of successful efforts to build trust, relevance, and connection with communities to promote community health malaria workers' acceptance. With lessons learned from the COVID-19 response, community health workers can be repurposed for broader public health interventions in preparation for future disease outbreaks.

## Teaser

**Key Findings:**

- Malaria Consortium-supported mobile malaria workers (MMWs) and mobile malaria posts reported no disruptions in services, based on comparisons between 2020 and the same month-period from the previous year.
- The communities trust the MMWs because they are recruited from within their communities and are often known to them personally, they speak the same language and are engaged in the same forest activities. Likewise, the MMWs trust their supervisors because of the clear duty of care displayed to them and the provision of consistent, relevant, and rapid support.
- A management approach that emphasized trust, relevance, and connection (TRC) made the communities and health service program more resilient to external factors beyond their control and ensured people feel comfortable delivering and using MMW services even during times of uncertainty, such as the COVID-19 pandemic.

**Key Implications:**

- If malaria elimination goals are to be successfully reached, it is vital to continue delivering essential early diagnostic and treatment services even during a time of potential crisis.
- TRC and flexible programming ensure communities and health services are resilient and less dependent on external factors, making it possible for essential service delivery to continue with minimal disruption.
- Scaling up the TRC approach to the wider MMW program will assist Cambodia, and potentially other settings, in achieving malaria elimination, regardless of the presence of COVID-19 or other potential extraneous disruptive events.

## INTRODUCTION

In 2011, Cambodia set an ambitious goal of the complete elimination of all *Plasmodium* malaria by 2025 in its National Strategic Plan,^1^ which was amended in 2016 with the Malaria Elimination Action Framework.^2^ Since then, the country's malaria elimination database shows impressive progress in reducing malaria trends from January 2018–May 2020. In 2018, no malaria-related deaths were reported for the first time.^3^

However, the coronavirus disease (COVID-19) pandemic presents a potential challenge to this goal. As observed in other countries around the world, COVID-19 can quickly overwhelm health system capacity and divert attention from other pre-existing health priorities. In African countries, the models predicted that the indirect effect of COVID-19 on intervention coverage would lead to additional cases and deaths in 2020 when compared to the previous years and possibly lead to further increases in subsequent years as a result of COVID-19 related disruptions to malaria control.^4^

**Figure:**
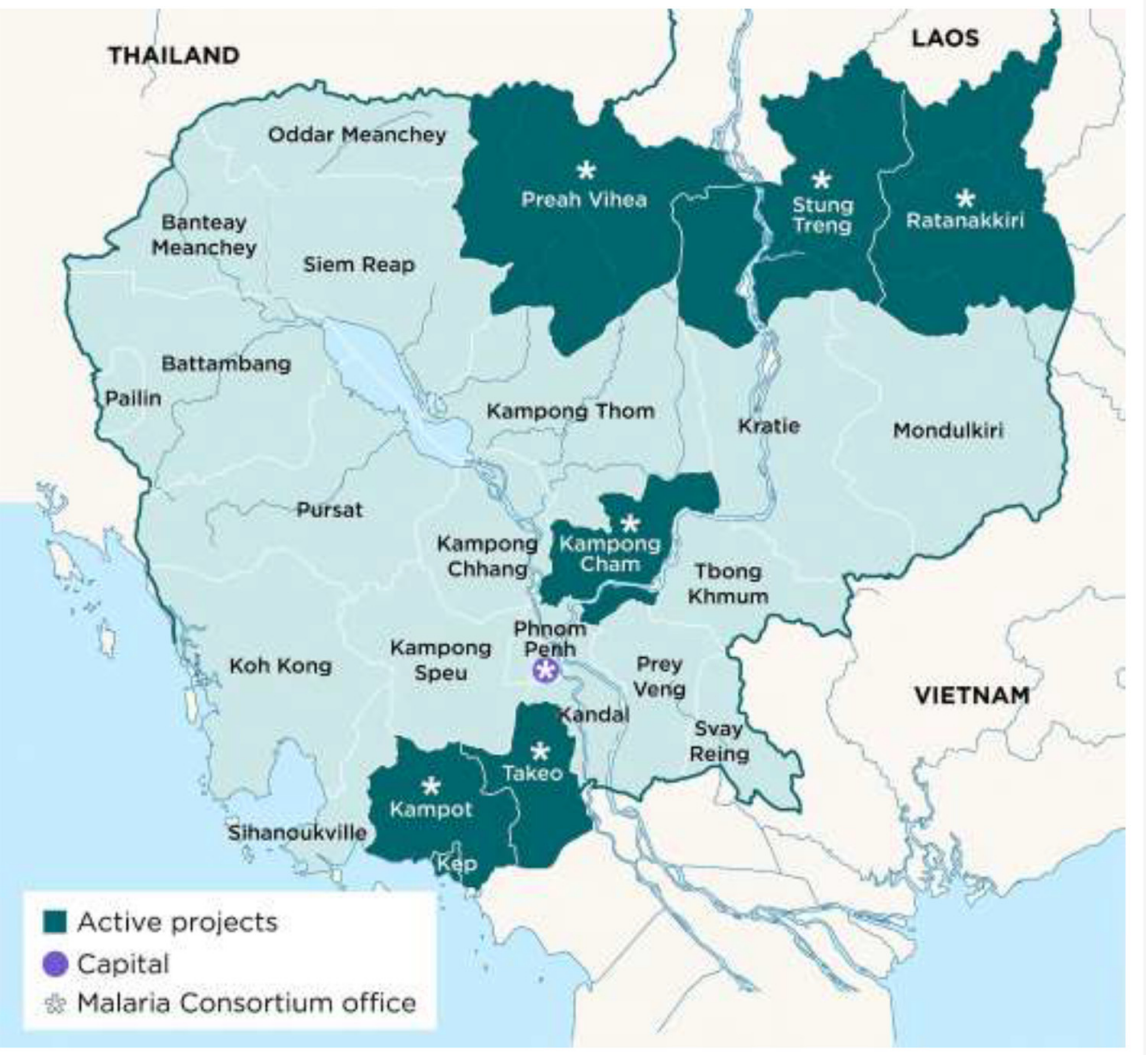
A mobile malaria worker in Cambodia provides health education at a malaria post. © 2020 Malaria Consortium Cambodia

As Peter Sands of The Global Fund said^5^:

*There's a significant risk that the knock-on consequences of COVID-19, in terms of the impact on other diseases, are likely to overshadow the direct impact.*

According to the World Health Organization (WHO), if COVID-19 significantly disrupts the primary health system, it's estimated that malaria deaths could double in sub-Saharan Africa and 80 million children could go unvaccinated for preventable but deadly diseases like measles or polio.^6^^,^^7^ Fortunately, Cambodia appears to have avoided this situation, and health service delivery was largely able to continue uninterrupted.

Situated relatively close to China, Cambodia was quickly on high alert as international news first reported the outbreak of COVID-19 in Wuhan and the subsequent lockdown of the city. Cambodia's first COVID-19 case was diagnosed on January 27, 2020, (unpublished data from WHO Cambodia Malaria Team and Mekong Malaria Elimination Programme) and the country responded over the next 2 months by setting up a national COVID-19 committee in March, reducing international travel and implementing screening points at border crossings to provide health education and fever screening for returning Cambodian migrant workers. Travel within the country continued except during the holiday period of Khmer New Year (April 2020), which is normally associated with high levels of social gathering and interaction. The Ministry of Health (MOH), WHO, and other partners ramped up preparations for the health sector to accommodate a potential increase in COVID-19 cases and developed an Emergency Master Plan for COVID-19 Response. The MOH updates the number and location of COVID-19 cases and deaths daily^8^ and posts daily surveillance reports.^9^ As of April 3, 2021, there have been a total of 1,041 COVID-19 cases and 91 deaths.^8^

In anticipation of the potential disruption on malaria intervention coverage and utilization due to indirect effects of the COVID-19 pandemic, Malaria Consortium set up an internal monitoring mechanism for the mobile malaria workers (MMWs) it supports, using performance output indicators, to detect any deviations from the planned target. Based on the interim findings, Malaria Consortium developed a COVID-19 contingency plan and made subsequent adjustments.

Anticipating the potential disruption on malaria intervention coverage and utilization due to the COVID-19 pandemic, Malaria Consortium developed a contingency plan and made subsequent adjustments.

## A COMMUNITY-BASED APPROACH TO INCREASE MALARIA SERVICE ACCESS

In 2009, the Cambodia National Center for Parasitology, Entomology, and Malaria Control (CNM) introduced a new cadre of community health workers (CHWs) known as MMWs to specifically improve the availability and accessibility of malaria services among remote populations (Box).^10^

BOX **Community Health Program in Cambodia**

CHWs provide a range of services in the communities they serve, such as health education and promotion, and ensuring community participation in health campaigns, etc.

Village malaria workers (VMWs) and mobile malaria workers (MMWs) are part of the broader cadre of community health workers (CHWs) that specifically support malaria activities for hard-to-reach communities. These CHWs are chosen by the community and are supported by either nongovernmental organization (NGOs) or the government but are not recognized as employees by either institution. They are provided with modest monetary incentives as compensation for their services. Using CHWs to deliver health services in their source communities has been shown to be effective in improving coverage of interventions, leading to reductions in mortality (unpublished data). However, in some settings, for example, where CHWs have not been carefully selected, appropriately trained, and adequately supported, having a high number of CHWs per population has not led to expected levels of improvement (unpublished data). Medicine and equipment shortages among CHWs or at facilities and poor quality of care in facilities also limit CHWs' ability to perform their duties and gain community trust.^8^

**Integrating and Institutionalizing CHWs Into the Health System**

One of the major drawbacks of the CHW program in Cambodia is that it was not properly institutionalized and integrated into the formal health system, has a vertical implementation nature, and relies on external financial and technical assistance. Their performance largely depended on the input (funding, commodity supply, and national policies), supportive systems (supervision, training, and incentives), and community support. In one instance, the Global Fund (a major donor for malaria activities in Cambodia) Office of Inspector General Audit noted in 2017 that due to delay in implementing fiduciary safeguards, VMW activities were suspended for a considerable period of time, which resulted in decline of service delivery.^11^ Following this instance, additional safeguarding measures were introduced by the Global Fund, including allowing NGOs to support the health centers, provincial health departments, and operational districts to manage the CHWs in a more effective and efficient manner.

In 2016, Cambodia placed a high value on the right to health for all Cambodians in its Health Strategic Plan 2016–2020 and adopted the mission of ^12^:

*effectively managing and leading the entire health sector to ensure that quality health services are geographically and financially accessible and socio-culturally acceptable to all people in Cambodia.*

Through health sector reform efforts, the Cambodian government has prioritized the expansion of health service coverage through the public sector (national to health center level) as well as via private-for-profit and private nonprofit organizations. Cambodia's MOH recognizes the important role nongovernmental organizations (NGOs) play in health service delivery, particularly through community-based health networks and coordination with the health institutions at all levels.

Malaria Consortium, along with other local and international NGOs, formed a partnership with CNM in Cambodia and complements CNM in delivering services to the communities as outlined in the goals of both the National Strategic Plan^1^ and Malaria Elimination Action Framework.^2^ The Global Fund and U.S. President's Malaria Initiative provide funding for the strategic plan. Collaboration between CNM and its implementation partners ensures compliance to national and donor rules and regulations and upholds accountability to avoid implementation delays encountered earlier.

The performance of village malaria workers (VMWs) and MMWs is closely monitored to ensure data and service quality. VMWs/MMWs are provided a modest monthly incentive based on their performance output, which is supported by internal data quality assurance and financial safeguarding mechanisms. Supervisors ensure data quality by checking and counter-checking data reported by MMWs for consistency, completeness, and accuracy against the daily case register books and also check other program-related data (e.g., stock, long-lasting insecticidal-treated net/long-lasting insecticidal-treated hammock net distribution, time and place of supervision visits, etc.). A community mobilization officer (CMO), which supervises VMWs/MMWs, or health center staff member also conducts routine supervision visits (once every month) to ensure data are of adequate quality.

### Building Trust and Acceptance Within the Community

In addition to the supportive systems (e.g., supervision and commodities), support from the community is key to maintaining high-quality VMW/MMWs' performance. Because they are recruited from within their communities, they are likely to have both a personal and a service relationship with the people they visit.^13^ Being a member of a community does not guarantee that CHWs will be trusted. Community perceptions of CHWs' motivation and competence shape people's willingness to communicate with and listen to CHWs, which in turn shapes CHWs' ability to fulfill the role of service extender, cultural broker, or social change agent.^14^ To be successful, not only do specific efforts have to be made to ensure trust among the communities and CHWs, but between the CHWs, health facility staff, and supervisors.^13^ For example, in Thailand, seeing CHWs work alongside public health professionals increased CHW credibility in the eyes of the community.^15^

In the case of the MMWs in Cambodia, those who have strong support and supervision from the CNM, and its partners, have similarly increased credibility within the communities they serve. As noted by Echaubard et al., genuine community engagement creates a sense of ownership ^16^:

*Motivated, empowered, and well-informed multistakeholder groups … should be better equipped to understand the tools available to them and mitigate cross-scale social and ecological drivers of disease emergence.*

MMWs who have strong support and supervision from the CNM, and its partners, have similarly increased credibility within the communities they serve.

Therefore, CHWs are better placed to identify and sustainably implement adaptive strategies. Early and effective community engagement, non-threatening home visits that enhance friendship, and strong supportive supervision can improve the trust and acceptance of the CHWs within the communities, as well as the confidence of the CHWs themselves, increasing the willingness of community members to use CHW services.^17^

Successful CHW programs require a partnership between communities and health systems (and MOH partners), however, this does not happen automatically. Explicit mutual responsibilities and accountabilities are required, as well as a demonstrated willingness to work in tandem toward a common objective and flexibility.^18^ To date, there have been limited examples showing how this collaborative, dynamic approach and trust-building effort can strengthen resilience and help maximize the efficient use of available resources.

## MOBILE MALARIA WORKER'S ROLE IN ELIMINATING MALARIA

To help Cambodia achieve its malaria elimination goal by 2025, Malaria Consortium, as one of CNM's implementing partners, supports the provision of early diagnostic and treatment services for malaria among remote populations through MMWs and mobile malaria posts (MPs) in 3 provinces in North East Cambodia (Preah Vihear, Stung Treng, and Ratanakiri Provinces). The approach was developed in alignment with the National Strategic Plan for Elimination of Malaria, in close collaboration with CNM, and built on lessons learned from earlier Regional Artemisinin-resistance Initiative (RAI) projects.^19^ The MMWs need to have a strong understanding of the local geography, since road access and river crossings change frequently, and work in collaboration with local authorities. Most importantly, MMWs need to build and maintain trust among the people who live in and around the forest, particularly because some may be involved in illegal activities and have cultural and linguistic differences from the majority Khmer population. It is essential that services to these communities are provided by a trusted and culturally acceptable person so that the communities use the services being provided. To achieve this trust, Malaria Consortium uses a peer-to-peer approach, with the majority of MMWs representing at least 2 of the following groups: forest goers, communities that regularly cross borders, loggers, ethnic minority groups, migrant farmers, or construction workers.

**FIGURE 1:**
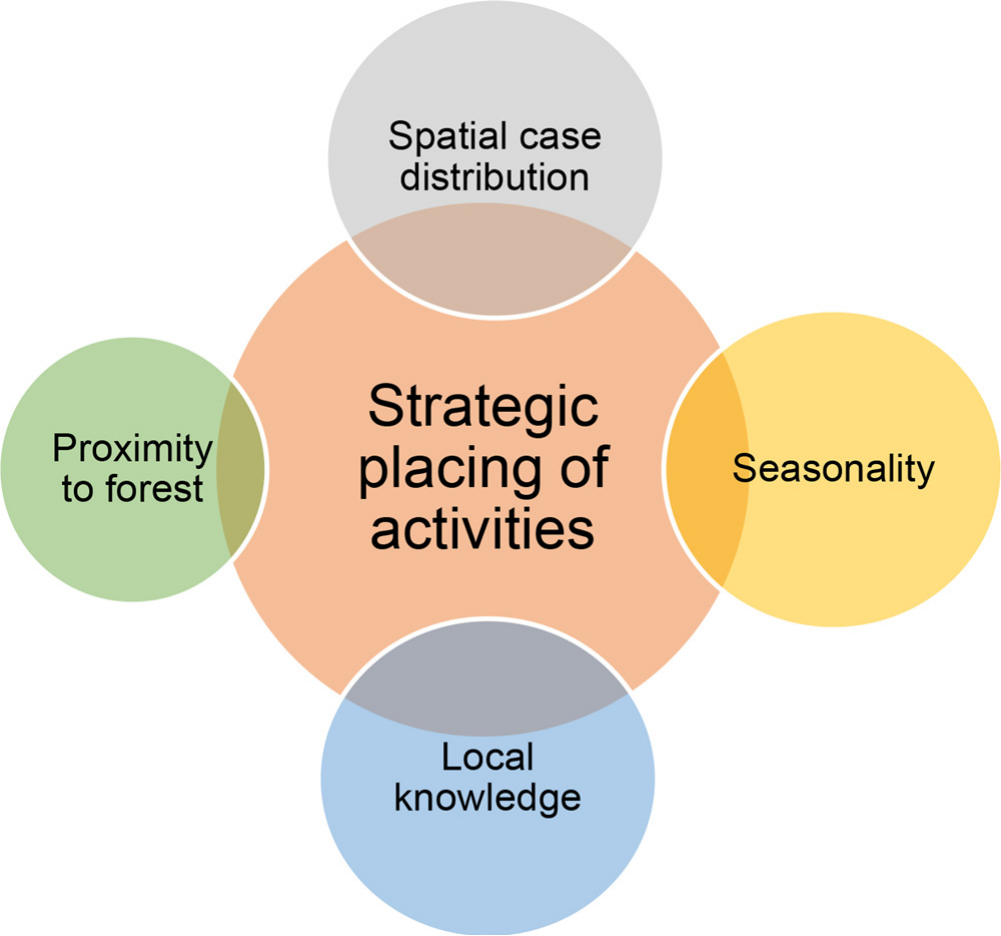
Map Showing Example of Reach of Malaria Consortium-supported Malaria Mobile Worker Service Delivery, Cambodia

The MMWs are trained and incorporated into the national VMW program and meet monthly with health center staff. This process ensures they are included in the general delivery of health services and can share challenges with health center staff. However, unlike traditional VMWs, who provide passive-case detection in their respective villages, MMWs provide active case detection by actively finding possible infections (whether symptomatic or not) that pose risks of infection and can cause onward transmissions. Active case detection targets at-risk populations, hard-to-reach, household members of positive cases, co-exposed, and co-travelers of the positive case who are usually less accessible to village-based testing or health centers. While doing so, MMWs adjust how they deliver their services, depending on changing circumstances and local epidemiology (Figure 1 shows an example of the reach of MMW service delivery).

MMWs provide active case detection by actively finding possible infections that pose risks of infection and can cause onward transmissions.

**FIGURE 2:**
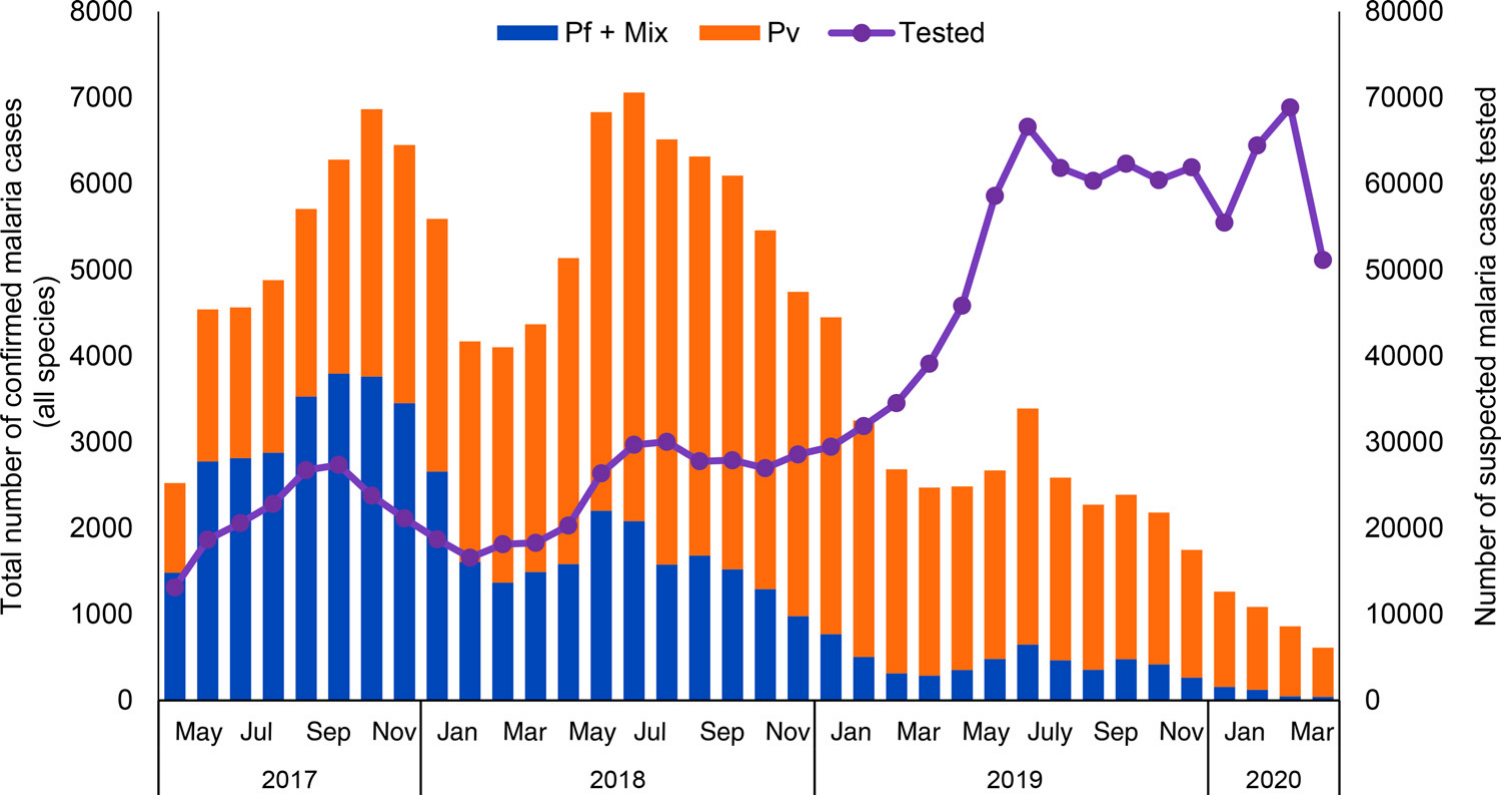
Triangulation Approach Malaria Consortium Used to Determine Where to Deliver Malaria Mobile Worker/Mobile Post Services in Cambodia

To maximize the reach of activities and increase the population able to receive services, locations for MP placement and targeted outreach activities are based on the triangulation approach of routinely collected data and local information (Figure 2): (1) local MMW knowledge on social gathering sites, forest entry/exit points which are known as strategic locations to best reach forest goers; (2) epidemiology—distribution of suspected and confirmed malaria cases (age-groups and sex); and (3) accessibility of hard-to-reach and remote areas.

To ensure the same level of trust operates between the MMWs and Malaria Consortium, CMOs are employed by Malaria Consortium to support an average of 6 or 7 MMWs or MPs each. This enables each CMO to provide consistent supervision and performance appraisal using a standardized supervision checklist that includes data quality (data entry, consistency, and accuracy), service quality (treatment provided in line with national protocols), stock balance, and level of testing compared to previous months, etc. Any issues that the CMOs identify during supervision are then followed up. If the CMO cannot join outreach activities for any reason, a home visit to the MMW or the MP is always planned, at least once a month. These supportive supervision visits are opportunities for both the CMO (the project staff) and the MMWs to review data and individual performance, progress, and areas to improve, as well as to develop plans for improvement. This 2-way problem-solving approach adds to the level of trust shared between the project staff and MMWs and subsequently increases the trust between MMWs and the communities. In addition, these processes ensure that MMWs sustain their motivation and job satisfaction, helping to reduce attrition.

## PROGRAM ADJUSTMENTS FOR COVID-19

National guidance documents and operational plans for malaria interventions were quickly adapted by CNM and WHO for the context of COVID-19 (unpublished data). Malaria Consortium's policy during COVID-19 has been to follow national guidelines while continuing to support community-based malaria services via the MMWs/MPs with as minimal disruption as possible. Malaria Consortium rapidly conducted a risk assessment and quickly developed and implemented a mitigation plan to ensure MMWs were able to continue providing services without putting themselves or their patients at risk (Table 1). Developed in consultation with the field-level project staff, community members, and the MMWs, the mitigation plan prioritized: the MMWs' safety and well-being during the pandemic (e.g., procuring personal protective equipment [PPE] and communicating on how to properly utilize and safely dispose of PPE); communication with the community members to address their risk perceptions around COVID-19 transmission and any fears or concerns they had related to receiving malaria services from the MMWs; program planning (in advance, quantification and distribution planning of long-lasting insecticidal-treated net requirements to avoid stock-outs); and continuous monitoring of the project data to identify and respond to any transmission outbreaks.

**TABLE 1.** Malaria Consortium COVID-19 Risk Assessment and Mitigation Plan for Mobile Malaria Worker Activities, Cambodia

Malaria Risks for Malaria Consortium MMW/Mobile Posts	Mitigation Measures	Implementation Status	Outcome
Risk of decreasing number of tests done by MMWs	Procured additional PPE material Strengthened messaging on importance of use of PPE strengthened	Distributed extra masks and forehead thermometers, March/April 2020	No decrease in number of tests observed In Stung Treng, some decrease in tests for April 2020, but this is yearly recurrent phenomenon due to Khmer New Year Starting up activities in fields near villages resulting in less forest activities by population
Miscommunication/ limited understanding of COVID-19 transmission and prevention	Distributed posters related to COVID-19 transmission and prevention (provided by PHD)	Distributed information on COVID-19 to all MMWs, March Gave strong messages to continue screening, implementing/following safety instructions	Low number of COVID-19 cases/no proven local transmission makes it easier for MMWs to continue the task. Malaria Consortium staff continues to support and visit the MMW/MMP in a safe way.
Fear feeling at MMWs/MMP/Malaria Consortium staff level	Provided additional PPE^a^ materials and communication about fears	Completed March/April with ongoing sharing information and updating by management team (e.g., repeat safety measures, weekly update mails task force, etc.)	No fear observed among MMWs/MMP and Malaria Consortium staff; no local transmission and no cases in the area gives feeling of safety As MMWs/MMP are locally recruited, no limit in movement when some villages/areas were closed for a few days. No limits in traveling for outreach activities.
Coverage LLIN/LLIHN	Ongoing distribution of LLIN/LLIHN Stock-out observed from March 2020	Requested additional LLIN/LLIHN to continue activity LLIN: out of stock on national level (refill 2021) LLIHN: received June 2020 order	Distribution of LLIHN ongoing by MMW/MMP No more LLIN: increased risk for plantation workers, for new settlements in forested areas, for new remote annex villages
Number of positive malaria cases	System in place: Inform health center of any *Plasmodium falciparum* positive cases for foci investigation Malaria Consortium team perform active case detection (=screening co-travelers)	Monitoring Malaria Consortium internal database; compare/update national Malaria Information System and exchange data monthly with CNM/WHO intensification plan for Stung Treng and Ratanakiri.	Decrease in the 3 areas of all types of malaria cases (end of dry season, starting rainy season) from January until May 2020

Abbreviations: CNM, National Center for Parasitology, Entomology and Malaria Control; LLIN, long-lasting insecticidal-treated net; LLIHN, long-lasting insecticidal-treated hammock net; MMP, mobile malaria post; MMW, mobile malaria worker; PHD, provincial health department; PPE, personal protective equipment; WHO, World Health Organization.

a PPE supplies provided included soap, hand sanitizer, masks, gloves, and thermometer guns.

**Figure:**
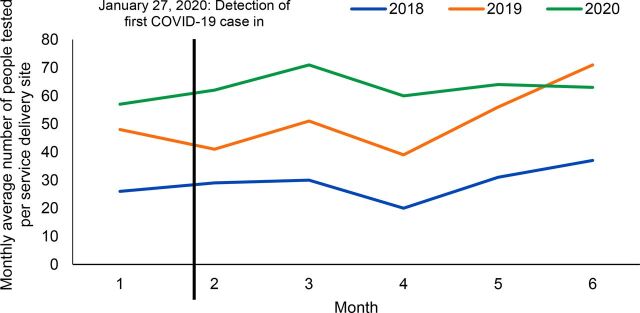
A mobile malaria worker in Cambodia provided with personal protective equipment and COVID-19 guidelines performs malaria testing on a forest-goer in Cambodia. © 2020 Malaria Consortium Cambodia

Performance monitoring mechanisms were set up and indicators have been closely monitored throughout the pandemic. MMW performance is monitored through quantitative indicators including average testing per service delivery site (e.g., outreach activity or MP), and test positivity rate (TPR) (Table 2 and Table 3). TPR was computed as the number of positive cases divided by the number of people tested in a specified period multiplied by 100.

**TABLE 2.** Average Number of People Tested Monthly per Service Delivery Site by Mobile Malaria Workers and Test Positivity Rate, From January to March, 2018–2020, Cambodia

	Average Monthly Active Sites, No.	Monthly Average Tested by Site, No.	Test Positivity Rate^a^ (All Plasmodium Species)	Test Positivity Rate^a^ (Pf/Mix)
January–March 2018	21	28	24.56%	13.6%
January–March 2019	45	47	7.23%	1.4%
January–March 2020	45	63	2.66%	0.2%

Abbreviation: Pf, Plasmodium falciparum.

a Test positivity rate calculated as the number of positive cases divided by number of tested in a specified period multiplied by 100.

**TABLE 3.** Average Number of People Tested Monthly per Service Delivery Site by Mobile Malaria Workers and Test Positivity Rate, From January to June, 2018–2020, Cambodia

	Average Monthly Active Sites, No.	Monthly Average Tested by Site, No.	Test Positivity Rate^a^ (All Plasmodium Species)	Test Positivity Rate^a^ (Pf/Mix)
January–June 2018	21	29	23.60%	12.2%
January–June 2019	45	51	5.35%	0.9%
January–June 2020	45	63	2.07%	0.1%

Abbreviation: Pf, Plasmodium falciparum.

a Test positivity rate calculated as the number of positive cases divided by number of tested in a specified period multiplied by 100.

## OUTCOMES

**FIGURE 3:**
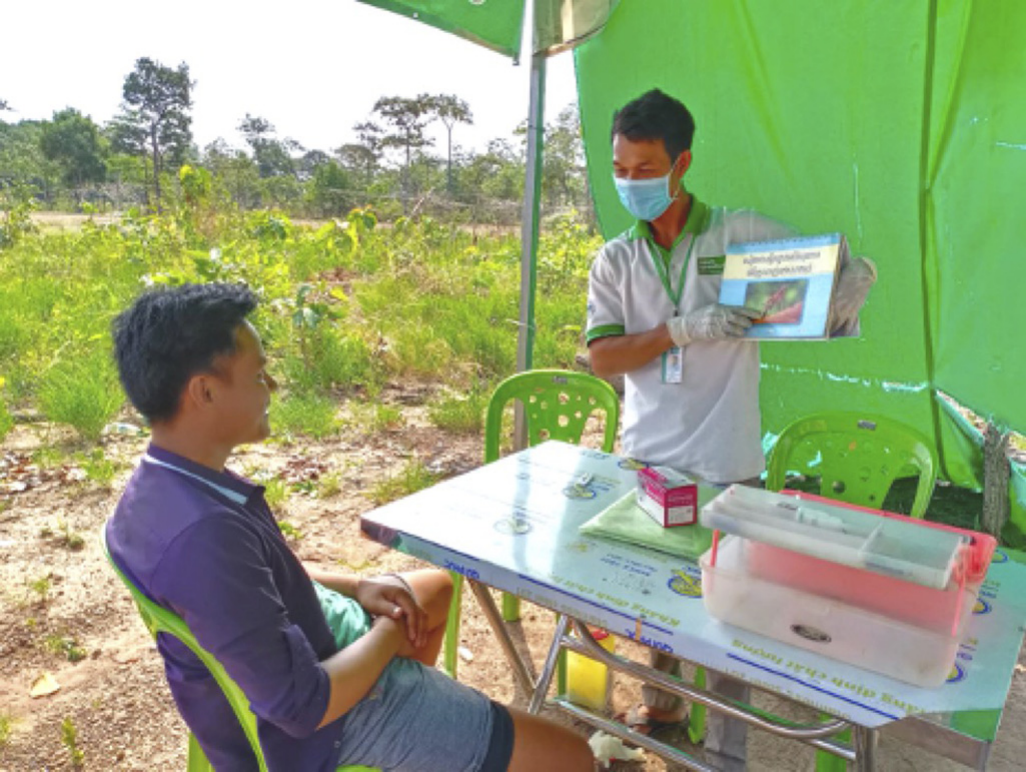
Malaria Testing and Confirmed Malaria Cases, in Cambodia, 2017–2020 Abbreviations: Pf, Plasmodium falciparum; Pv, Plasmodium vivax. Source: World Health Organization (WHO). Mekong Malaria Elimination: Epidemiology Summary July-September 2020. Vol. 11, WHO; 2020. Accessed April 5, 2021. https://www.who.int/publications/i/item/WHO-UCN-GMP-MME-2020.04

**FIGURE 4:**
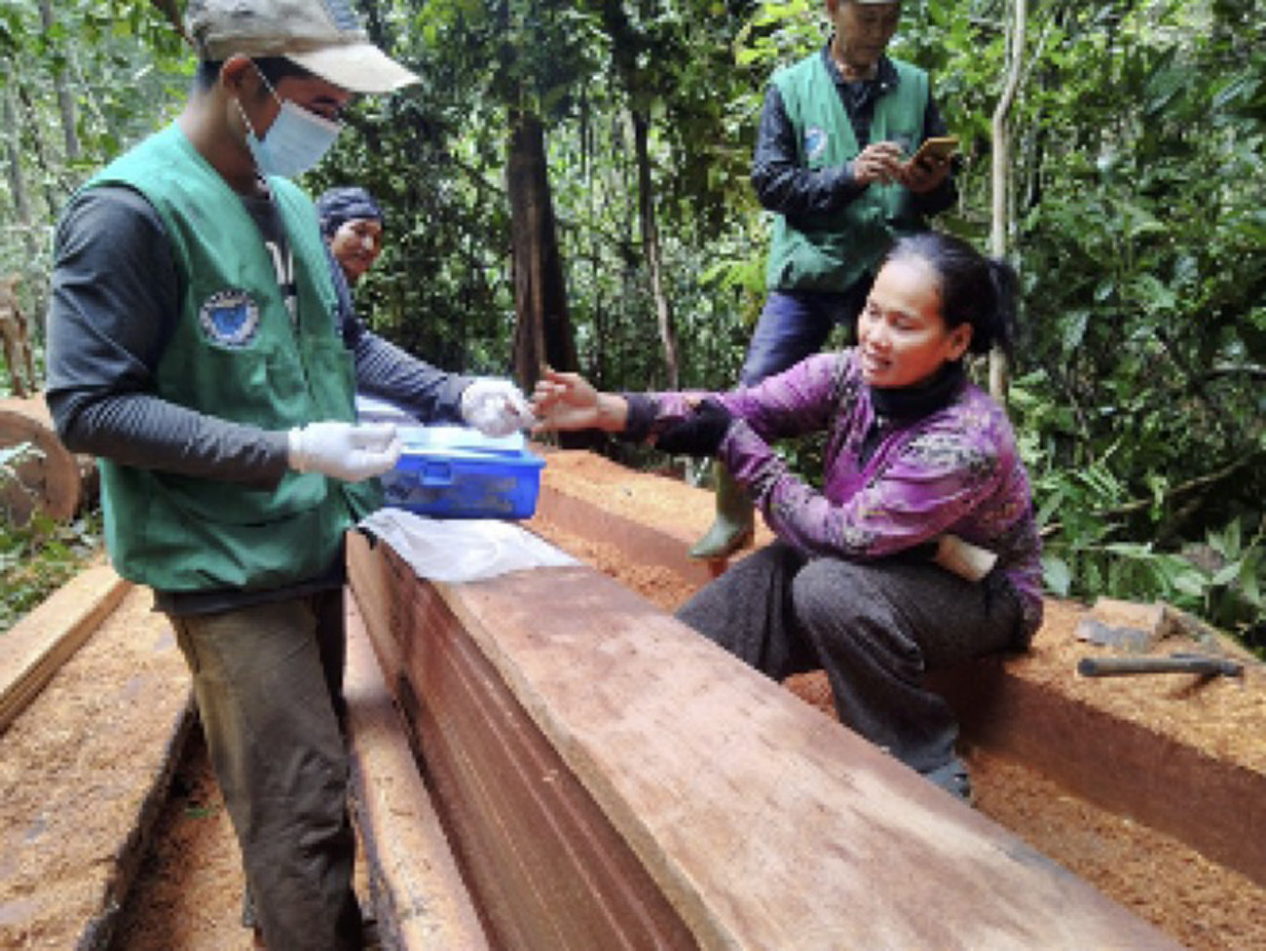
Malaria Testing Rates Done by Mobile Malaria Workers in Project Areas Between January and June 2018-2020, Cambodia

The reported number of confirmed COVID-19 cases has remained low in Cambodia. There have been only minor disruptions to health services. However, the number of malaria tests conducted nationally decreased by 20% in April and May compared to March (unpublished data). To date, there have been confirmed COVID-19 cases in Preah Vihear but not in the other 2 provinces supported by Malaria Consortium. But, as with the rest of the country, there has been an overall decline in malaria testing at the health centers and VMWs among all 3 provinces, according to WHO statistics. Such a decline was not seen in previous years and may be attributed to fear of COVID-19 (Figure 3 shows subregional testing and confirmed *Plasmodium falciparum* and mixed cases). However, based on a review of routine project data, the indicators suggest that there has been no decline in the utilization of malaria testing/treatment by Malaria Consortium-supported MMWs. During the pandemic period under review (January 2020–June 2020), the monthly average malaria testing rate was higher than in the same period in 2019 and 2018. The overall malaria TPR in Malaria Consortium supported areas continued to decline in 2020, which is consistent with the national disease trend (Figure 3). In fact, contrary to the initially anticipated program disruption and service utilization decline due to COVID-19, there were consistently high malaria testing rates of an average of 63 people per site sustained throughout from January–June 2020 in follow-up RAI2-Elimination project areas (Figure 4). One MMW commented:

*For sure, the activities of the MMWs in this area could still continue because the MMWs are all “recognized” and trusted by the local people. … CMOs and MMWs have heard information from people in the communities that some villagers were worried about getting infected by COVID-19. But they still come for malaria testing at the MPs or with the MMWs when they suspect they might have malaria, as they trust our services and clearly understood that COVID-19 can be prevented by wearing masks and washing their hands with soap or gel.* —MMW, Chom Ksant District, Preah Vihear Province

Another MMW reported the same experience:

*At the beginning of the outbreak of COVID-19, CMOs and MMWs heard some information from local people saying they were worried about the disease and afraid to go outside or to go to the town. They asked their children to stay at home. But when they suspect that they might get malaria, they will still go and meet with MMWs in the village, that they have known.* —MMW, Cham Village, Siem Pang District, Stung Treng Province

## LESSONS LEARNED

Malaria Consortium ensured the MMW/MP program is built on trust, relevance to, and connection within the communities being served. Realization of these philosophies requires sound programmatic processes and measurements of the MMWs' performance and timely proactive mitigation planning. Community systems are fragile and vulnerable to disruption particularly at the time of international health crises. Community access to services was not disrupted because the services continued to be available within their communities and eliminated the need to travel a long distance just to access care, often at their own expense. Accessing the MMWs without diverting to any other providers requires a level of trust.

The communities trust the MMWs because the MMWs are part of the community, are known personally by community members, speak the same language, and are engaged in the same forest activities. Providing quality supportive supervision to MMWs, actively communicating and engaging with the community to address their perceived risks during the pandemic, prioritizing the MMWs' well-being and safety by providing PPE, and regularly communicating with MMWs on their day-to-day issues were all processes that culminated in making the community health systems more resilient to external factors beyond their control. These processes also ensured that, even during times of uncertainty, such as the COVID-19 pandemic, MMWs were comfortable delivering services and communities were comfortable using MMW and MP services.

If malaria elimination goals are to be successfully reached, it is vital to continue delivering essential early diagnostic and treatment services even during a time of potential crisis. Building community resilience through trust, relevance, and connection and using flexible programming ensure communities and health services are less dependent on external factors, making it possible for essential service delivery to continue with minimal disruption. Scaling up this supportive approach to the MMW program could allow Cambodia, and potentially other settings, to ensure they succeed in achieving malaria elimination, regardless of the presence of COVID-19 or other potential extraneous disruptive events.

If malaria elimination goals are to be successfully reached, it is vital to continue delivering essential early diagnostic and treatment services even during a time of potential crisis.

## KEY STEPS FOR ENSURING TRUST, RELEVANCE, AND CONNECTION

- Recruit MMWs directly from the communities they will serve
- Ensure quality support is provided by the CMOs (e.g., limit network size of MMWs/MPs supported by each CMO)
- Ensure flexibility with locations of MPs and outreach services through triangulation of relevant information
- Respond rapidly to changing circumstances
- Provide clear information and guidelines on changing situation (e.g., COVID-19 transmission and prevention)
- Ensure the safety of staff (both CMOs and MMWs) and those using services (e.g., rapid provision of PPE materials)
- Continue to provide visible support to MMWs despite changing circumstances

## CONCLUSIONS

Malaria Consortium was able to provide care and support for the MMWs and the communities being served by conducting a COVID-19 rapid risk assessment and mitigation plan, providing correct information on the transmission and prevention of COVID-19 and enhanced PPE, and continuing routine support supervision visits. The high level of trust already established by the program ensured a willingness among the MMWs and communities to continue providing and using services as usual.

The project's routine reports and preliminary feedback from the community suggest that Malaria Consortium's trust, relevance, connection strategy, programmatic processes, and proactive mitigation planning has been successful in reducing the indirect effect of the COVID-19 pandemic on the intervention coverage and service utilization by the community across the 3 supported provinces. This clearly demonstrates the important role of building and sustaining genuine trust among the communities served and service providers for the continuation of vital malaria elimination services, regardless of the ongoing external factors, such as COVID-19 or other future pandemics or natural disasters.

## Notes

**Peer Reviewed**

**First published online:** May 13, 2021.

## References

[1] Kingdom of Cambodia, Ministry of Health (MOH). National Strategic Plan for the Elimination of Malaria 2011–2025. MOH; 2011. https://www.cnm.gov.kh/userfiles/file/N%20Strategy%20Plan/National%20Strtegyin%20English.pdf

[2] Kingdom of Cambodia, Ministry of Health (MOH). Cambodia Malaria Elimination Action Framework 2016–2020. MOH; 2016. http://mesamalaria.org/sites/default/files/2018-12/Cambodia%20Malaria%20Elimination%20Action%20Framework%20.pdf

[3] World Health Organization (WHO). World Malaria Report 2019. WHO; 2019. Accessed April 3, 2021. https://apps.who.int/iris/rest/bitstreams/1262394/retrieve

[4] Weiss DJ Bertozzi-Villa A Rumisha SF. Indirect effects of the COVID-19 pandemic on malaria intervention coverage, morbidity, and mortality in Africa: a geospatial modelling analysis. Lancet Infect Dis. 2021;21(1):59–69. 10.1016/S1473-3099(20)30700-3. 32971006 PMC7505634

[5] Community health workers are critical to fight COVID-19—but they need PPE. Last Mile Health Blog. Accessed March 2, 2021. https://lastmilehealth.org/2020/06/26/chws-critical-to-fight-covid-19-need-ppe/

[6] World Health Organization. WHO urges countries to move quickly to save lives from malaria in sub-Saharan Africa. Accessed March 2, 2021. https://www.who.int/news/item/23-04-2020-who-urges-countries-to-move-quickly-to-save-lives-from-malaria-in-sub-saharan-africa

[7] World Health Organization. At least 80 million children under one at risk of diseases such as diphtheria, measles and polio as COVID-19 disrupts routine vaccination efforts, warn Gavi, WHO and UNICEF. Access March 2, 2021, https://www.who.int/news/item/22-05-2020-at-least-80-million-children-under-one-at-risk-of-diseases-such-as-diphtheria-measles-and-polio-as-covid-19-disrupts-routine-vaccination-efforts-warn-gavi-who-and-unicef

[8] Kingdom of Cambodia, Ministry of Health. Total number of COVID infections. Accessed April 3, 2021. https://covid19-map.cdcmoh.gov.kh/

[9] Kingdom of Cambodia, Ministry of Health, Communicable Disease Control Department. COVID-19 daily surveillance reports. Accessed April 3, 2021. http://cdcmoh.gov.kh/resource-documents/daily-surveillance-reports

[10] Canavati SE Quintero CE Lawford HLS. High mobility, low access thwarts interventions among seasonal workers in the Greater Mekong Sub-region: lessons from the malaria containment project. Malar J. 2016;15(1):434. 10.1186/s12936-016-1491-3. 27562347 PMC5000443

[11] The Global Fund, Office of Inspector General (OIG). Audit Report: Global Fund Grants in the Kingdom of Cambodia. Global Fund; 2017. Accessed April 6, 2021. https://www.theglobalfund.org/media/6761/oig_gf-oig-17-020_report_en.pdf

[12] Kingdom of Cambodia, Ministry of Health (MOH). Third Health Strategic Plan 2016-2020. MOH; 2016. Accessed April 3, 2021. http://hismohcambodia.org/public/fileupload/carousel/HSP3-(2016-2020).pdf

[13] Grant M Wilford A Haskins L Phakathi S Mntambo N Horwood CM. Trust of community health workers influences the acceptance of community-based maternal and child health services. Afr J Prim Health Care Fam Med. 2019(1):e1–e8. 10.4102/phcfm.v9i1.1281. 28582988 PMC5458568

[14] Schaaf M Warthin C Freedman L Topp SM. The community health worker as service extender, cultural broker and social change agent: a critical interpretive synthesis of roles, intent and accountability. BMJ Glob Health. 2020;5(6):e002296. 10.1136/bmjgh-2020-002296. 32546585 PMC7299037

[15] Kowitt SD Emmerling D Fisher EB Tanasugarn C. Community health workers as agents of health promotion: analyzing Thailand's village health volunteer program. J Community Health. 2015;40(4):780–788. 10.1007/s10900-015-9999-y. 25744815

[16] Echaubard P Thy C Sokha S. Fostering social innovation and building adaptive capacity for dengue control in Cambodia: a case study. Infect Dis Poverty. 2020;9(1):126. 10.1186/s40249-020-00734-y. 32883345 PMC7469325

[17] Singh D Cumming R Negin J. Acceptability and trust of community health workers offering maternal and newborn health education in rural Uganda. Health Educ Res. 2015;30(6):cyv045. 10.1093/her/cyv045. 26459326

[18] Naimoli JF Perry HB Townsend JW Frymus DE McCaffery JA. Strategic partnering to improve community health worker programming and performance: features of a community-health system integrated approach. Hum Resour Health. 2015;13(1):46. 10.1186/s12960-015-0041-3. 26323276 PMC4556219

[19] Malaria Consortium. Trans-border Malaria: Mapping High-risk Populations and Targeting Hotspots With Novel Intervention Packages. Malaria Consortium; 2017. Accessed April 3, 2021. https://www.malariaconsortium.org/resources/publications/743/trans-border-malaria-mapping-high-risk-populations-and-targeting-hotspots-with-novel-intervention-packages

